# Utilization of biosynthesized silver nanoparticles from *Agaricus bisporus* extract for food safety application: synthesis, characterization, antimicrobial efficacy, and toxicological assessment

**DOI:** 10.1038/s41598-023-42103-3

**Published:** 2023-09-12

**Authors:** Maryam Amr, Samah H. Abu-Hussien, Radwa Ismail, Asmaa Aboubakr, Rahma Wael, Mariam Yasser, Bahaa Hemdan, Salwa M. El-Sayed, Ashraf Bakry, Naglaa M. Ebeed, Hesham Elhariry, Ahmed Galal, Basma T. Abd-Elhalim

**Affiliations:** 1 Undergraduate student, New Programs, Faculty of Agriculture, Ain Shams University, PO Box 68, Hadayek Shoubra, Cairo, 11241 Egypt; 2 Department of Agricultural Microbiology, Faculty of Agriculture, Ain Shams University, PO Box 68, Hadayek Shoubra, Cairo, 11241 Egypt; 3 Water Pollution Research Department, Environment and Climate Change Research Institute, National Research Centre, 33 El-Bohouth St., Dokki, Giza, 12622 Egypt; 4 Department of Biochemistry, Faculty of Agriculture, Ain Shams University, PO Box 68, Hadayek Shoubra, Cairo, 11241 Egypt; 5 Department of Genetics, Faculty of Agriculture, Ain Shams University, PO Box 68, Hadayek Shoubra, Cairo, 11241 Egypt; 6 Department of Food Science, Faculty of Agriculture, Ain Shams University, PO Box 68, Hadayek Shoubra, Cairo, 11241 Egypt; 7 Department of Poultry Production, Faculty of Agriculture, Ain Shams University, PO Box 68, Hadayek Shoubra, Cairo, 11241 Egypt

**Keywords:** Biotechnology, Microbiology

## Abstract

The emergence of antimicrobial resistance in foodborne bacterial pathogens has raised significant concerns in the food industry. This study explores the antimicrobial potential of biosynthesized silver nanoparticles (AgNPs) derived from *Agaricus bisporus* (Mushroom) against foodborne bacterial pathogens. The biosynthesized AgNPs were characterized using various techniques, including UV–visible spectroscopy, X-ray diffraction, Fourier transform infrared spectroscopy, high-resolution scanning electron microscopy with energy dispersive X-ray spectroscopy, dynamic light scattering, and zeta potential analysis. The antibacterial activity of the AgNPs was tested against a panel of foodborne bacterial strains, and their cytotoxicity was evaluated on normal human skin fibroblasts. Among the tested strains, Pseudomonas aeruginosa ATCC 27853 showed the highest sensitivity with an inhibition zone diameter (IZD) of 48 mm, while Klebsiella quasipneumoniae ATTC 700603 and Bacillus cereus ATCC 11778 displayed the highest resistance with IZDs of 20 mm. The silver cations released by AgNPs demonstrated strong bactericidal effects against both Gram-positive (G + ve) and Gram-negative (G − ve) bacteria, as evidenced by the minimum inhibitory concentration/minimum bactericidal concentration (MBC/MIC) ratio. Moreover, cytotoxicity testing on normal human skin fibroblasts (HSF) indicated that AgNPs derived from the mushroom extract were safe, with a cell viability of 98.2%. Therefore, AgNPs hold promise as an alternative means to inhibit biofilm formation in the food industry sector.

## Introduction

The food sector faces critical problems due to bacterial resistance towards antibiotics, chemical cleansers and disinfectants. Foodborne pathogens affect the healthcare settings, as they can cause foodborne illnesses in patients such as Salmonellosis, Shigellosis, Toxoplasmosis, Viral gastroenteritis, Taeniasis and Trichinosis^1,2^. Therefore, finding new alternatives for improving different disinfectant methods against bacterial pathogens is necessary^3^.

Nanomaterials such as AgNPs offer promising techniques for controlling pests, improving human health, and enhancing various industrial sectors, including agriculture and energy^4,5^. AgNPs have been known for their strong antibacterial activity for several decades, and many researchers have studied the antimicrobial activities of AgNPs in combination with different phytochemicals against pathogenic bacteria and fungi^6,7^. However, it is essential to note that using AgNPs and other nanomaterials also raises concerns about their potential toxicity and environmental impact^8^. Nano materials cytotoxicity varies depending on many different factors, including their size, shape, surface area, and surface chemistry^9^. Therefore, there is a need to carefully evaluate the potential risks and benefits of using nanomaterials in various applications^10^. Despite these challenges, using nanomaterials in multiple fields shows excellent potential for addressing global challenges such as food security, energy production, and healthcare^4^. Ongoing research and development in biotechnology field would be critical for harnessing their full potential and minimizing their potential risks as well as ensuring their safety use^11^.

Recently, the biosynthesized nanoparticles have attained high recognition due to their many advantages over chemically and physically synthesized nanoparticles^11,12^. Biosynthesized nanoparticles are nontoxic, safe for humans, eco-friendly, and economically valuable^13–15^. They are also known for their antimicrobial properties, as they could stop the growth of multidrug-resistant bacteria^16^. One of the key advantages of biosynthesized nanoparticles is that they can be produced using simple and cost-effective methods, such as biological and green synthesis methods^17^. For example, microorganisms, plants, and fungi can synthesize nanoparticles, which can be applied in many fields such as medicine, agriculture, and environmental remediation^4,18^. The biosynthesized nanoparticles have been shown to have antimicrobial activity against a wide range of bacterial, fungal, and viral strains^19,20^. They can also be used as drug delivery systems^21^, as they can target specific cells and tissues in the body^22^. Additionally, biosynthesized nanoparticles have been shown to have potential in various environmental applications, such as water treatment and pollution control^23^. The characterization of the biosynthesized AgNPs can provide valuable information on their size, shape, and stability, which can affect their antimicrobial activity^24^. The antimicrobial potential of the AgNPs can be evaluated using various methods, such as disc diffusion and minimum inhibitory concentration assays^20,25,26^.

Using plant and microbial metabolite extracts to promote the bio-reduction of Ag^+^ and produce biosynthesized AgNPs is a promising approach^17^. Phytochemicals and microbial metabolites can act as reducers, stabilizers, and capping agents in biosynthesis^22^. mushrooms are a rich source of medicinal compounds, including polysaccharides, terpenoids, glucans, phenolic compounds, statins, and lectins^24^. These natural precursor compounds play an important role in reducing silver ions into AgNPs with high stability and good dispersion characteristics. In addition to the main objective mentioned above, this research aims to delve deeper into the synthesis and characterization of silver nanoparticles (AgNPs) derived from *A. bisporus* mushrooms. The synthesis process involves utilizing a liquid extract obtained from *A. bisporus* mushrooms as a precursor for AgNP synthesis. The researchers will carefully control the reaction conditions to ensure the successful formation of AgNPs. Following the synthesis, we conducted a comprehensive characterization of the AgNPs. This characterization process involves analyzing various physical and chemical properties of the AgNPs, such as size, shape, surface characteristics, and stability. Techniques such as microscopy, spectroscopy, and other analytical methods were employed to understand the AgNPs' characteristics thoroughly. Furthermore, we evaluated the antimicrobial efficacy of the synthesized AgNPs against different foodborne pathogenic bacteria. The AgNPs were tested against various bacterial strains known to cause foodborne illnesses. The antimicrobial activity was assessed using appropriate methods, such as agar diffusion assays or minimum inhibitory concentration (MIC) determination. Moreover, Cytotoxicity of HSF was detected.

## Materials and methods

### Chemicals and reagents

Silver nitrate (AgNO_3_) was purchased from Sigma, Aldrich, Germany and was used for silver solution prepration. Standard antibiotics (Ampicillin and Streptomycin,1000 µg/mL) were purchased from Amoun pharmaceutical company**,** Cairo, Egypt. All chemicals were analytical grade.

### *Agaricus bisporus* source and aqueous extract preparation

Fresh *A. bisporus* fruiting bodies were collected from local hypermarkets in Cairo, Egypt, wrapped in plastic bags and transferred to Microbiology lab. at the Faculty of Agriculture, Ain Shams University, Cairo, Egypt. Preservation of mushroom was carried out by cooling process at 4 °C till using. For AgNPs extraction, All fruiting bodies were thoroughly cleansed with deionized water (DW). Twenty five grams of *A. bisporus* were cut into tiny pieces and cooked in glass beakers containing 100 mL of DW for 2 h using a magnetic stirrer hot plate. The extract was filtrated using Whatman No. 1 filter paper. All collected filtrates was maintained at 4 °C for further studies^27^.

### Pathogenic microbial strains

In current study, eight pathogenic bacterial strains, namely: *Bacillus cereus* ATCC 11778, *Escherichia coli* ATCC 8379, *Enterococcus faecalis* ATCC 29212, *Klebsiella quasipneumoniae* ATTC 700603, *Pseudomonas aeruginosa* ATCC 27853, *Salmonella typhi* DSM 17058, *Shigella sonnei* DSM 5570 and *Staphylococcus aureus* ATCC 29737 were obtained from Agric. Microbiology Department, Faculty of Agriculture, Ain Shams University, Cairo, Egypt.

### Standard inoculum

Nutrient broth medium (NBM) was prepared in Erlenmeyer flask (250 mL) containing 50 mL of the NBM and inoculated by a single pure colony of the tested bacterial culture^28^. All inoculated flasks were incubated at 30 °C at 150 rpm for 24 h. One milliliter of this culture had 4.5–5.77 × 10^5^ colony forming unit (CFU/mL)^17^. All experimental trials were carried out in triplicates.

### Biosynthesis of AgNPs from *A. bisporus*

For AgNPs biosynthesis, aqueous silver nitrate (AgNO_3_) solution and aqueous mushroom extract was prepared to reduce Ag^+^ to Ag^0^ to produce AgNPs. To initiate biosynthesis, 10 mL of aqueous mushroom extract is added to 90 mL solution of the the silver nitrate (0.1 M). This mixture is then mixed well and incubated in a rotary shaker incubator (Shin Saeng, South Korea) overnight at an agitation speed of 150 rpm in the dark at room temperature. A negative control involved mushroom extract without adding silver nitrate solution^29^. The color of the mushroom extract is observed to turn brown, indicating the formation of AgNPs.

### Characterization techniques of the biosynthesized AgNPs from *A. bisporus*

The surface images of nanoparticles were obtained using HR-SEM–EDX (VEGA 3-T Scan, Brno, Czech Republic) image resolution 512 by 442, image pixel size: 0.04 μm, with a 20.0 kV accelerating voltage. The FTIR analysis has been subjected to AgNPs samples on Bruker VERTEX 80 (Germany), in the wavenumber range 4000–400 cm^−1^, with resolution 4 cm^−1^, refractive index 2.4. The stability of AgNPs was analyzed by measuring the absorbance using UV–Vis (JASCO Corp., V-570 USA) spectrum at 200–800 nm. The crystalline states of nanoparticles were obtained using powder XRD analysis using X-ray diffractometer (Shimadzu -7000, United Kingdom). The size distribution of particles and zeta potential charge was estimated via DLS using Zetasizer Nano Series (HT), Nano ZS, Malvern Instruments, UK. The DLS was used to provide information about the AgNPs properties at “long wavelength” limited by the wavelength of light^30^. Zeta potential was measured as one of the characteristics of charged colloids that may be determined using phase analysis light scattering based on DLS and laser Doppler velocimetry , with count rate 286 kHz, and wavelength (λ = 1.54 nm) at 30 °C and viscosity 0.995cp^31^.

### Antibacterial activity of the biosynthesized AgNPs from *A. bisporus*

#### Evaluation of antimicrobial activity using agar well-diffusion assay

Mueller–Hinton agar plates were prepared, 1 mL of the standard inoculum of each tested bacterial culture containing 4.5–5.77 × 10^5^ CFU/mL was added onto the agar surface, individually. Wells were made by using a sterile cork porer (8 mm) in diameter, 100µL of 1000 µg/mL AgNPs were added into wells^6^ using ampicillin and streptomycin (1000 mg/mL) as positive control for G − ve and G + ve bacteria, respectively. The plates were then incubated at 37 °C for 24 h. The antimicrobial activity was assayed by measuring the IZD in millimeters (mm) around the growth of colonies. To evaluate the antimicrobial activity of the AgNPs, the activity index was calculated^32^ by comparing the diameter of the inhibition zone of AgNPs with the standard reference antibiotic.

#### Assessment of minimum inhibitory concentration (MIC)

MIC assay was performed according to the method recommended by the Clinical and Laboratory Standard Institute^33^. Two-fold serial dilutions of the AgNPs were prepared, starting from a concentration of 1000 µg/mL (control) and decreasing to concentrations of 500, 250, 125, 75, 50, 25, and 12.5 µg/mL. Inoculation was done on MHA as previously described. All plates incubated at 37 °C for 24 h. MIC was determined as the lowest concentration of AgNPs at which no visible bacterial growth was observed.

#### Assessment of minimum bactericidal concentration (MBC)

Based on the results of MIC, MBC was calculated by sub-culturing negative results of MIC test by subculturing MHA medium and incubating at 37 °C for 24 h. The lowest concentration of AgNPs that showed no growth on MHA medium was recorded as the MBC value.

#### Assessment of AgNPs mode of action

MBC/MIC ratio was calculated^34^, ratios of value ≥ 4 or greater was considered as a bactericidal effect able to kill the bacterial cells. The MBC/MIC ratio of value ≤ 2 showing a bacteriostatic effect able to inhibit the bacterial growth.

### Cytotoxicity assay of AgNPs from *A. bisporus*

Cytotoxicity of the AgNPs was assessed using the 3-(4,5-dimethylthiazol-2-yl)-2,5-diphenyltetrazolium bromide (MTT) assay. Briefly, DMEM media with supplemented 100 mg/mL of streptomycin, 100 U/mL of penicillin, and 10% of heat-inactivated fetal bovine serum was prepared and kept at a humidified, 5% (v/v) CO_2_ atmosphere then incubated at 37 °C. Aliquots of 100μL cell suspension containing 5 × 10^3^ cells were seeded in 96-well plates and incubated in complete media for 24 h at 37 °C in 5% CO_2_. Cells were then treated with various concentrations of AgNPs for 48 h. After drug exposure, the media was discarded, and the MTT solution (20μL of 1 mg/mL stock solution) was added to 100μL of phosphate buffer solution (PBS) in each well and incubated at 37 °C for 4 h. The formazan crystals formed by the live cells were then dissolved with 100μL of absolute dimethyl sulfoxide (DMSO)^9^. The absorbance of formazan solutions was measured at λmax 570 nm using a micro-plate reader (Synergy-HT, BioTek) to estimate % viability^35^.

### Statistical analysis

All samples and collected data were statistically analyzed using IBM^®^ SPSS^®^ Statistics software (2017). Tukey test at a *P*-value of 0.05 was applied^36^.

### Ethical statement

This article does not contain any studies with human participants or animals performed by any of the authors.

## Results

### Biosynthesis of AgNO_3_ using *Agaricus bisporus*

**Figure 1:**
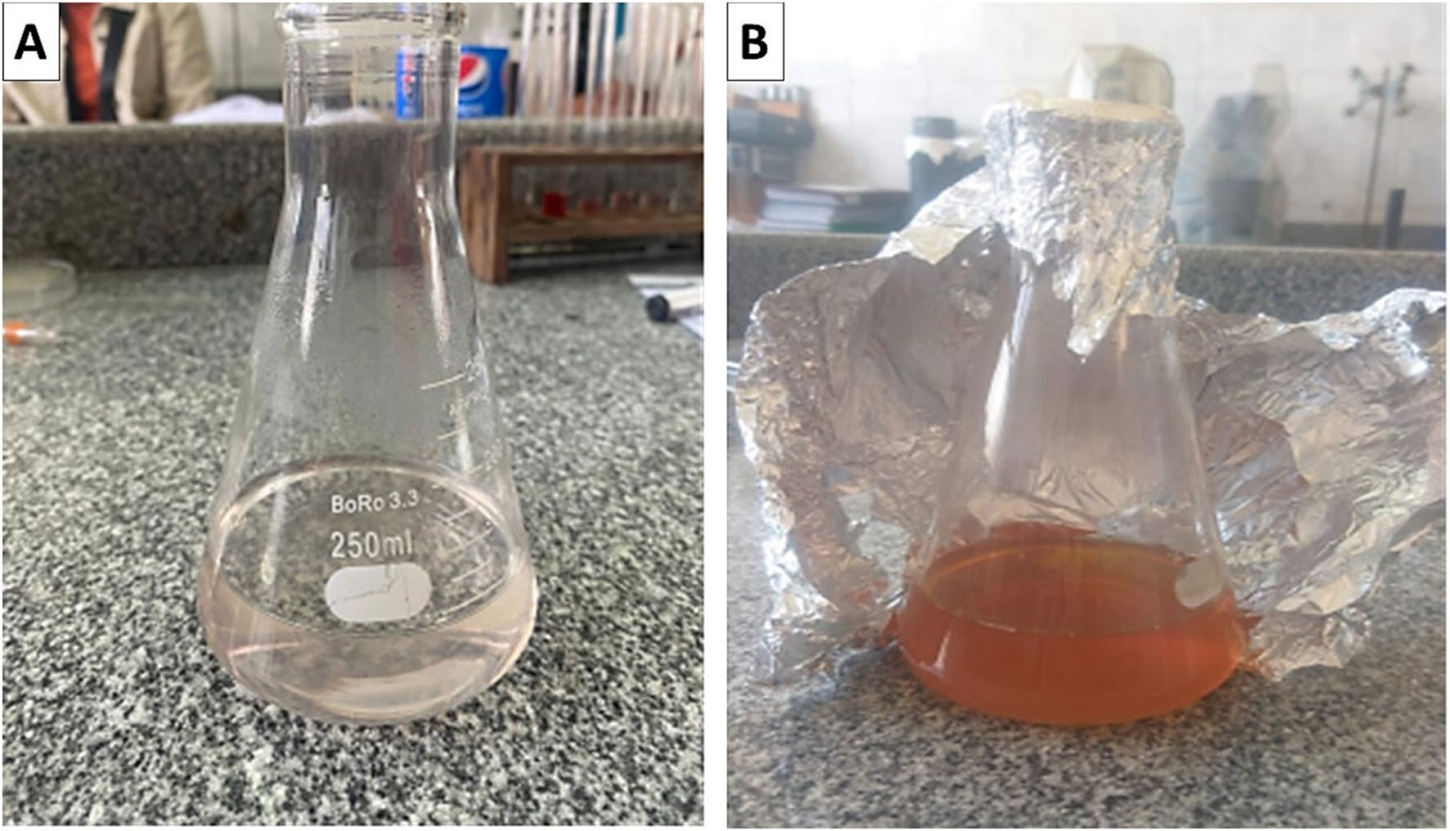
Color variations before and after the conversion of Ag^+^ to Ag^0^ nanoparticles. (**A**) Mushroom extract with no silver nitrate (AgNO_3_) (negative control). (**B**) mushroom extract with added silver nitrate (AgNO_3_).

In the current study, 10 mL of the silver nitrate (0.1 M) solution is added to 90 mL of the aqueous mushroom extract and incubated in a rotary shaker overnight to synthesize AgNPs biologically. Gradually change in color was observed from pale yellow color (before the reduction of Ag^+^) to a brownish solution (after the reduction of Ag^+^), indicating the formation of AgNPs due to the excitation of nanoparticles’ SPR. The mushroom extract incubated with deionized water (negative control) maintained its original color, as shown in Fig. 1.

### Characterization of the biosynthesized AgNPs from *A. bisporus*

#### UV–Vis spectroscopy analysis

**Figure 2:**
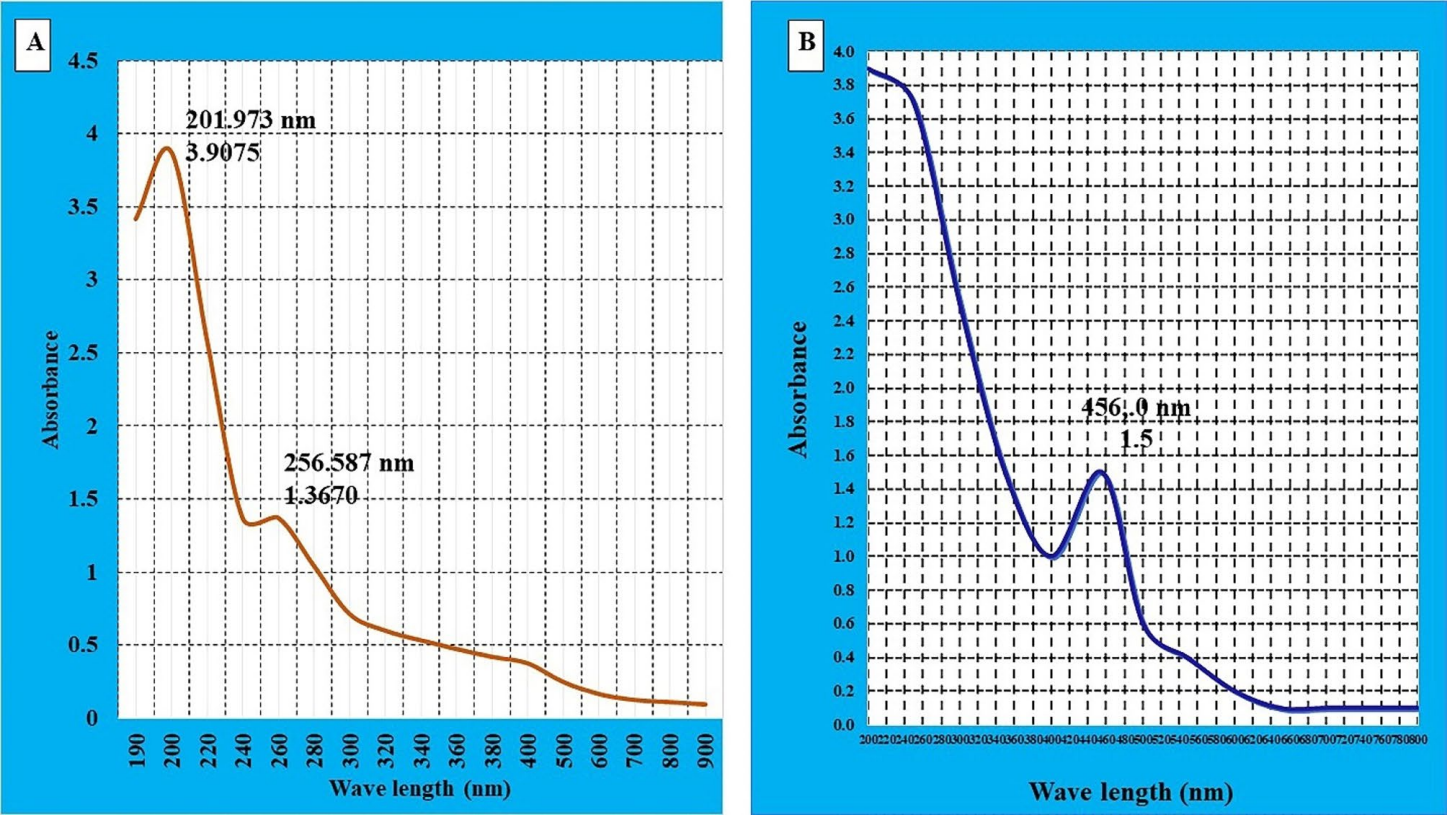
UV–Vis spectrum of biologically synthesized AgNPs from *A.bisporus* aqueous extract. (**A**) control treatment, (**B**) AgNPs from mushroom extract.

The UV–Vis spectroscopy analysis of both control and AgNPs mushroom extracts were characterized in a comparison as shown in Fig. 2. The Uv–Vis spectrum of control extract and of *A. bisporus* was detected at 265.5 nm with O.D of 1.37, while the AgNPs surface plasmon band was detected at 456 nm with SPR of 1.5.

#### X-Ray diffraction (XRD)

**Figure 3:**
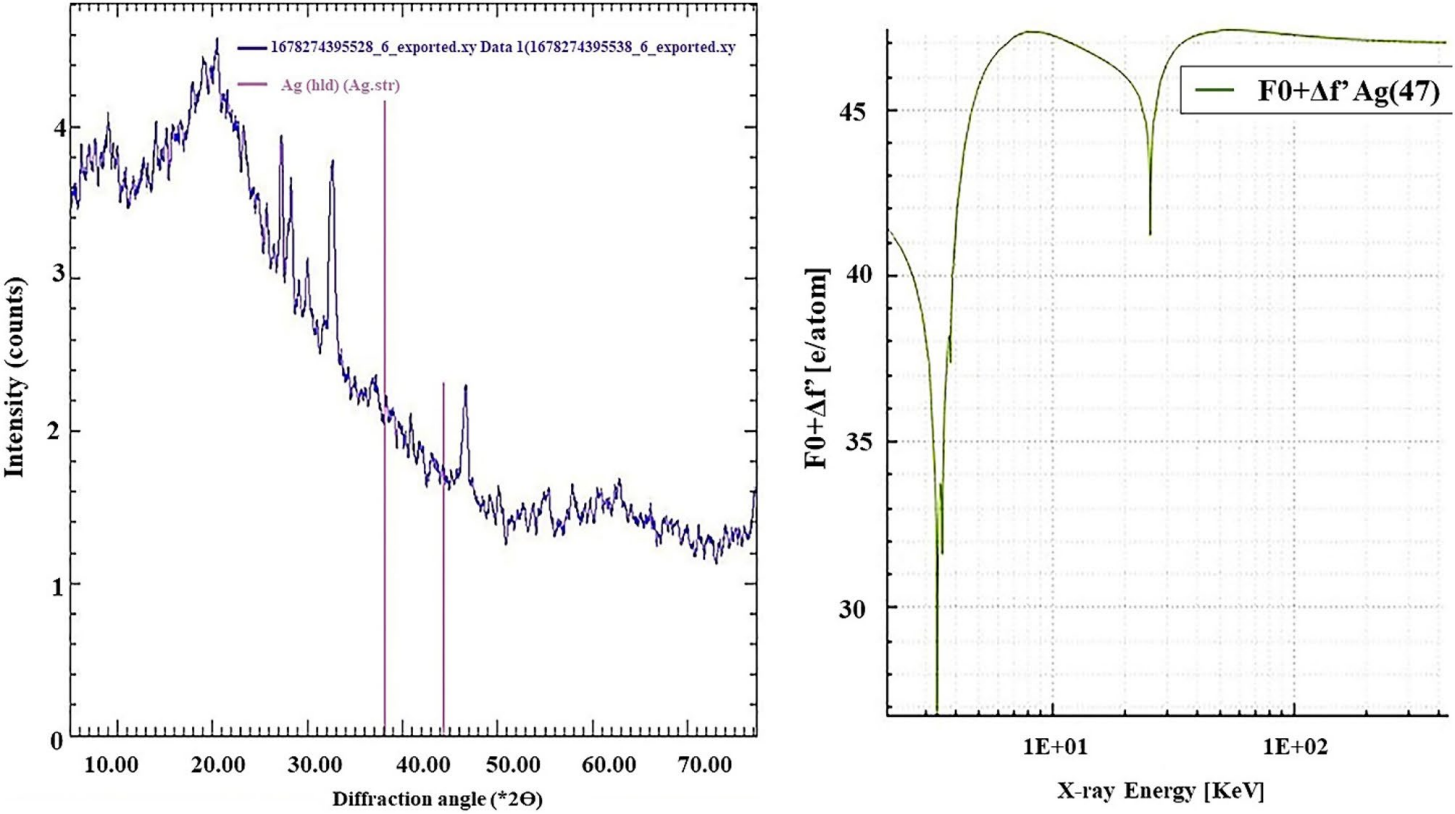
X-ray diffraction (XRD) pattern of biosynthesized AgNPs from *A. bisporus*.

X-ray diffraction (XRD) graphs were plotted in Fig. 3 to study the crystalline nature and lattice characteristics of the biosynthesized AgNPs. Diffraction intense peaks were observed at 2θ values at 38.5°, and 45.5° corresponding to planes (111), and (200), respectively.

#### High-resolution scanning electron microscopy with energy dispersive X-ray spectroscopy (HR-SEM–EDX)

**Figure 4:**
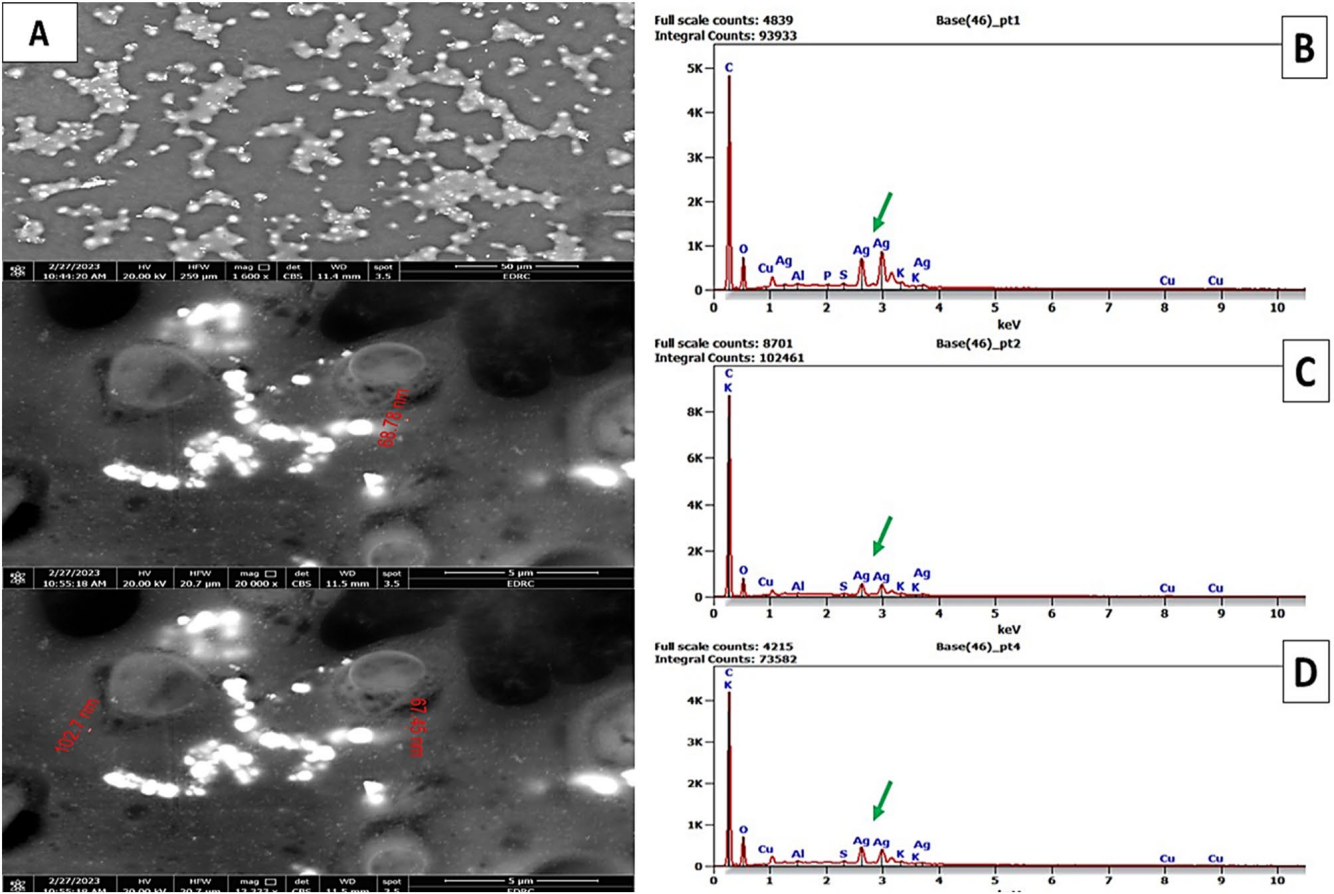
Scanning Electron Microscope (SEM) and EDX images indicating the biosynthesized AgNPs from *A.bisporus*.

SEM–EDX technique was used to define both the nanoparticle shape and size of the biosynthesized AgNPs. From the obtained images, AgNPs were quasi-spherical shaped, and their size ranged from 67.45 to 102.7 nm. It also can be noticed from the SEM images in Fig. 4 that the bio-synthesized AgNPs are well stabilized and capped by active compounds present in the *A. bisporus* extract. The EDX spectrum of AgNPs displayed a strong peak at ∼2 keV suggests the formation of AgNPs succefully.

#### Fourier transform infrared spectroscopy (FTIR) analysis

**Figure 5:**
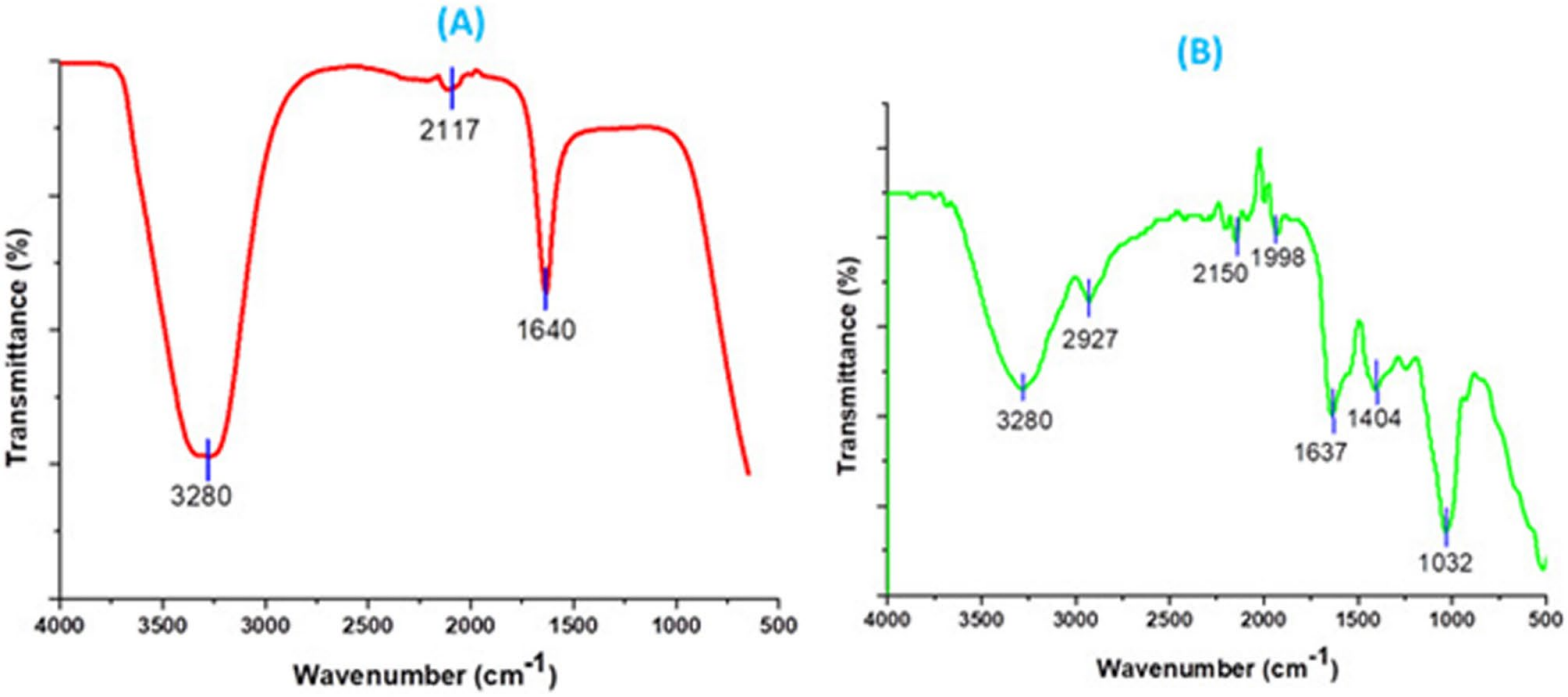
FTIR spectrum of biosynthesized AgNPs from *A. bisporus* by reduction of Ag^+^ ions. (**A**) before AgNPs biosynthesis, (**B**) after the AgNPsbiosynthesis.

The FTIR characterization analysis of the control *A. bisporus* extract compared to the biosynthesized AgNPs was illustrated in Fig. 5. The formation of silver nanoparticles can be confirmed by the presence of a peaks at 514 and 1032.09 cm^−1^ belong to bending vibration of Ag.

#### Dynamic light scattering (DLS)

**Figure 6:**
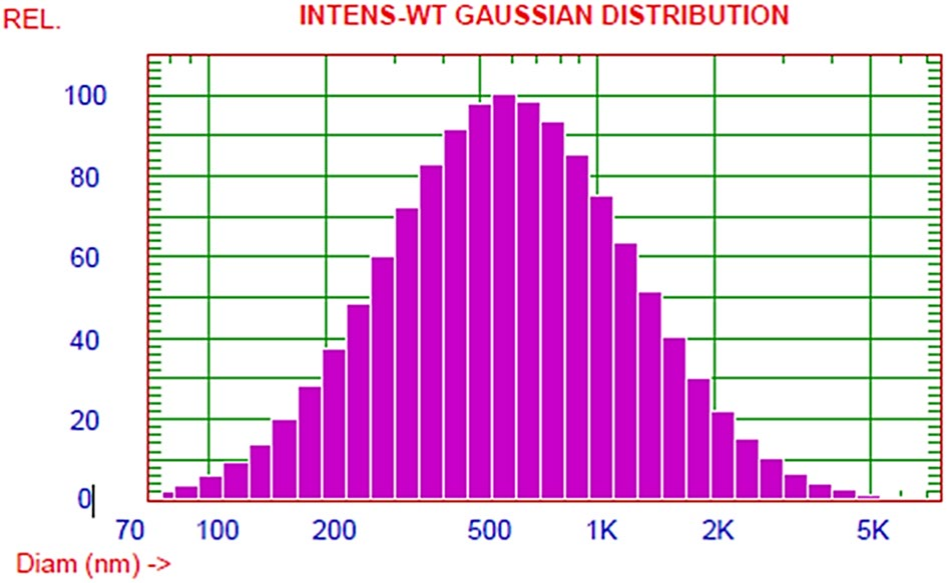
Dynamic light scattering (DLS) results for AgNPs from *A. bisporus*.

In the current study, the mean diameter of the biosynthesized nanoparticles was 765.7 nm as shown in Fig. 6.

#### Zeta potential

**Figure 7:**
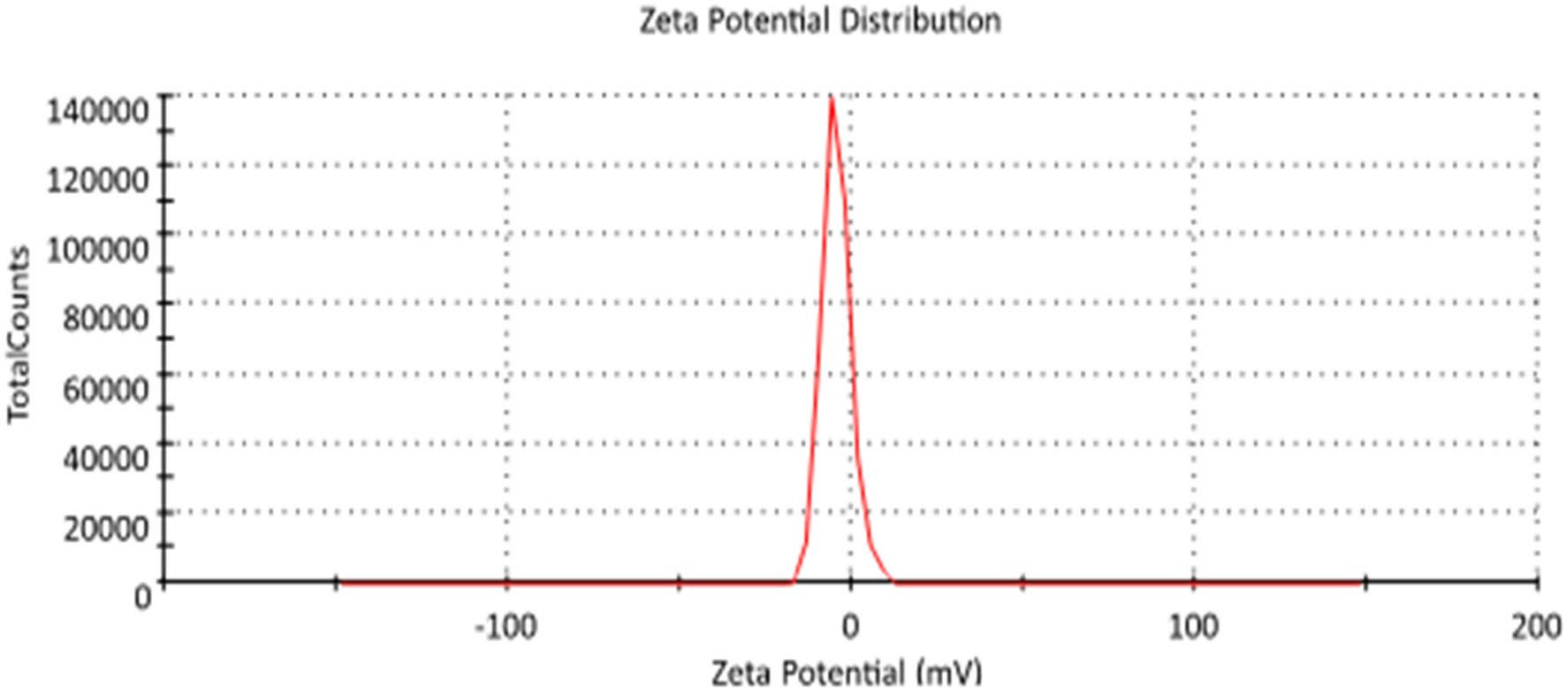
Zeta potential record for AgNPs from *A. bisporus*.

AgNPs stability can be easily determined by recording Zeta Potential (ξ) value. The ξ value recorded for AdNPs was − 5.77 mV as shown in Fig. 7. The negative value of ξ is seems due to the capping effect of the AgNPs by the phytochemicals present in the *A. bisporus* extract.

### Inhibitory activity of biosynthesized AgNPs from *A. bisporus* against foodborne pathogenic bacteria

**Figure 8:**
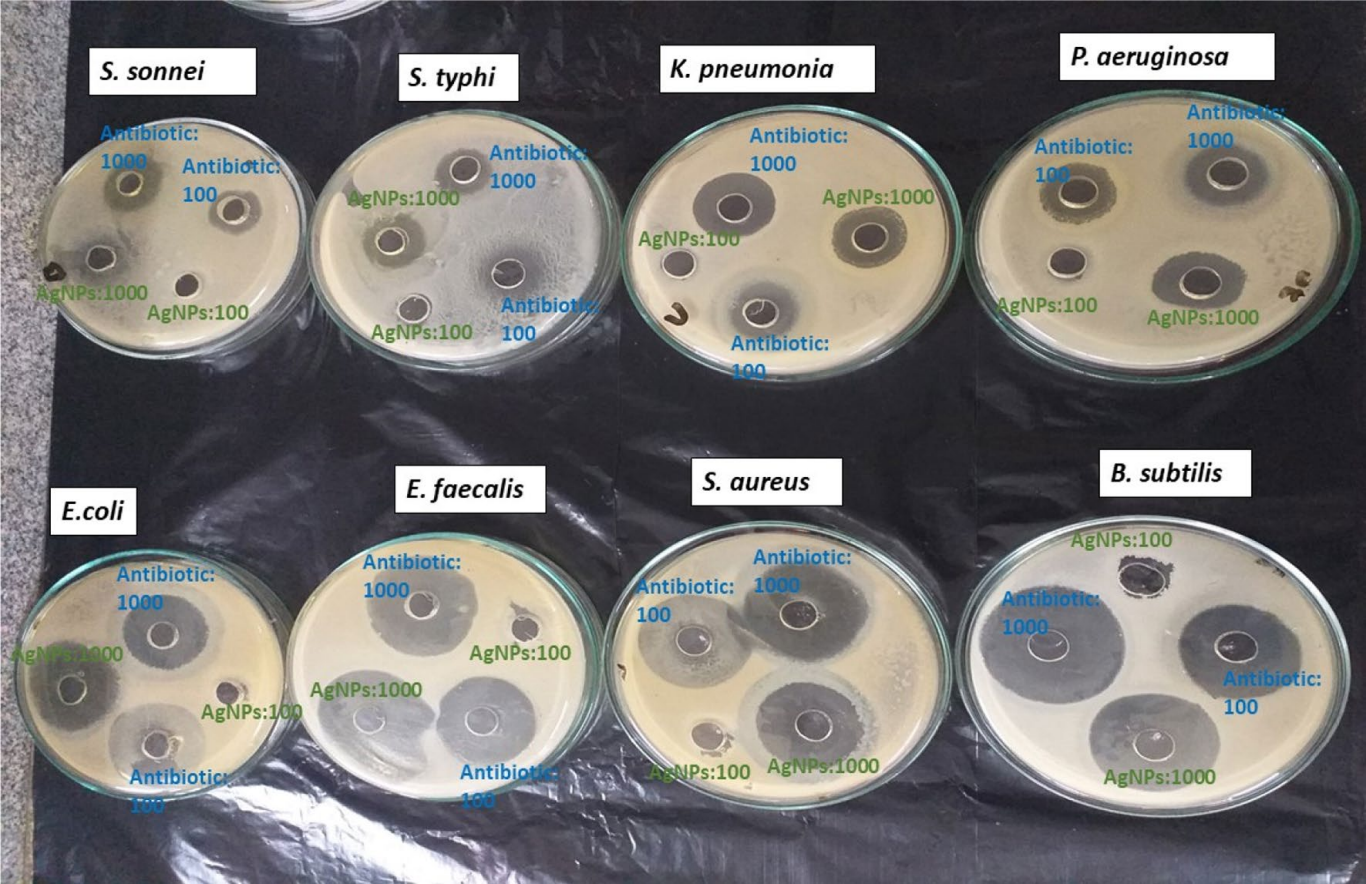
Antibacterial potential of biosynthesized AgNPs from *A. bisporus* against foodborne pathogenic bacteria (*S. sonnei*, *S. typhi*, *K. quasipneumoniae*, *P. aeruginosa*, *E. coli*, *E. fecalis*, *S. aureus* and *B. cereus*).

Results in Fig. 8 and Table 1 illustrated that *B. cereus*, *E. faecalis*, S. aureus were less sensitive to the biosynthesized AgNPs from *A. bisporus* with inhibition zones diameters (IZD) of 41.0, 28.0, and 20.0 mm, and Activity index (AI) of 1.33, 1.08, 1.00, respectively. *P. aeruginosa* showed intermediate resistance towards the biosynthesized AgNPs from *A. bisporus*. On the other hand, *S. typhi*, *E. coli*, *S. sonnei*, and *K. quasipneumoniae* were highly sensitive to the biosynthesized AgNPs from *A. bisporus* with IZD of 48.0, 30.0, 25.0, 24.0, 20.0 mm, respectively and AI of 0.69, 0.75, 0.69, 0.68, 0.70.

**Table 1:** Inhibition zone diameter (IZD) and activity index (AI) of biosynthesized AgNPs from *A. bisporus* against food born pathogenic bacterial strains.

Foodborne pathogenic strains	IZD (mm)		AI
	Antibiotic (1000 µg/mL)	AgNPs (1000 µg/mL)	
G^+ve^ Bacteria			
* B. cereus* ATCC 11778	30.0^ h^ ± 0.40	20.0^ m^ ± 0.50	1.33
* E. faecalis* ATCC 29212	49.0^a^ ± 0.86	41.0^d^ ± 0.41	1.08
* S. aureus* ATCC 29737	45.0^c^ ± 0.87	28.0^i^ ± 0.30	1.00
G^−ve^ Bacteria			
* E. coli* ATCC 8379	38.0^e^ ± 0.36	25.0^ k^ ± 0.14	0.69
* K. quasipneumoniae* ATTC* 700603*	27.0^j^ ± 0.85	20.0^ m^ ± 0.77	0.70
* P. aeruginosa* ATCC 27853	35.0f. ± 0.83	48.0^b^ ± 0.02	0.69
* S. typhi* DSM 17058	31.0^ g^ ± 0.22	30.0^ h^ ± 0.11	0.75
* S. sonnei* DSM 5570	35.0f. ± 0.24	24.0^ l^ ± 0.30	0.68

Standard antibiotics were streptomycin and ampicillin against G^+ve^ bacteria and G^−ve^ bacteria, respectively.

*mm* millimeter, *AI* activity index, *SE (* ±*)* standard error.

Values in the same column followed by the same letter do not significantly differ, according to Tukey’s at 5% level.

### Minimum inhibition concentration (MIC) of the biosynthesized AgNPs from *A. bisporus*

The results presented in Table 2 indicate that the minimum inhibitory concentration (MIC) values of AgNPs against the tested pathogenic bacterial strains ranged from 1000 to 12.5 μg/mL. The MIC value of 1000 μg/mL was observed for *E. coli, K. quasipneumoniae*, and *P. aeruginosa*, indicating that a higher concentration of AgNPs is required to inhibit the growth of these bacterial strains. The MIC value of 500 μg/mL was observed for *B. cereus, E. faecalis, S. typhi*, and *S. sonnei*, indicating that a lower concentration of AgNPs is required to inhibit the growth of these bacterial strains compared to *E. coli, K. quasipneumoniae*, and *P. aeruginosa*. The MIC value of 250 μg/mL was observed for *S. aureus*, indicating that the growth of this bacterial strain can be inhibited by a relatively lower concentration of AgNPs compared to the other bacterial strains tested. The results suggest that the antibacterial activity of AgNPs is concentration-dependent. At a concentration of 1000 μg/mL, the AgNPs showed 100% antibacterial activity against all the tested bacterial strains. At 500 and 250 μg/mL concentrations, the antibacterial activity was 62.5% and 12.5%, respectively. At concentrations of 125–12.5 μg/mL, no antibacterial activity was observed.

**Table 2:** MIC of AgNPs against clinical pathogenic bacterial strains after incubation at 37 °C for 24 h.

Foodborne pathogen strains	MIC (µg/mL) of AgNPs							
	1000 (control)	500	250	125	75	50	25	12.5
*B. cereus* ATCC 11778	_	_	+	** + **	** + **	** + **	+	** + **
*E. coli* ATCC 8379	_	+	** + **	** + **	** + **	+	** + **	+
*E. faecalis* ATCC 29212	_	_	** + **	** + **	** + **	** + **	+	** + **
*K. quasipneumoniae* ATTC 700603	_	+	+	+	** + **	** + **	+	** + **
*P. aeruginosa* ATCC 27853	_	** + **	** + **	** + **	** + **	** + **	+	** + **
*S. typhi* DSM 17058	_	_	+	** + **	** + **	** + **	+	** + **
*S. sonnei* DSM 5570	_	_	+	** + **	** + **	** + **	+	** + **
*S. aureus* ATCC 29737	_	_	_	+	** + **	** + **	+	** + **
Spectrum of activity (%)	8/8	5/8	1/8	0/8	0/8	0/8	0/8	0/8
	100	62.5	12.5	0	0	0	0	0

_no growth, + positive growth.

Overall, the results suggest that AgNPs have the potential as antibacterial agents against a broad range of pathogenic bacterial strains, with the antibacterial activity being dependent on the concentration of AgNPs. However, further studies are needed to evaluate the safety and efficacy of AgNPs for use as antibacterial agents.

### Minimum bactericidal concentration (MBC) of the biosynthesized AgNPs from* A. bisporus*

Values of MBC for AgNPs is presented in Table 3. The MBC values of the biosynthesized AgNPs from *A. bisporus* ranged from 1000 to 12.5 μg/mL. The MBC value was exhibited 1000 μg/ml for* E. coli*, *K. quasipneumoniae*, and *P. aeruginosa*, *B. cereus*, and* E. faecalis*. At the same time, it was 500 μg/mL with *S. typhi*, *S. aureus*, and* S. sonnei*. The results clearly show 100% of the antibacterial spectrum activity of AgNPs at concentration of 1000 μg/mL, whereas at a concentration of 500 μg/mL the activity was 37.5%. Whereas at concentrations of 250–12.5 μg/mL the antibacterial spectrum showed no activity.

**Table 3:** MBC of the biosynthesized AgNPs from *A. bisporus* against foodborne bacterial strains after incubation at 37 °C for 24 h.

Foodborne pathogen strains	MBC (µg/mL) of AgNPs							
	1000 (control)	500	250	125	75	50	25	12.5
*B. cereus* ATCC 11778	_	+	+	** + **	** + **	** + **	+	** + **
*E. coli* ATCC 8379	_	+	** + **	** + **	** + **	+	** + **	+
*E. faecalis* ATCC 29212	_	+	** + **	** + **	** + **	** + **	+	** + **
*K. quasipneumoniae* ATTC* 700603*	_	+	+	+	** + **	** + **	+	** + **
*P. aeruginosa* ATCC 27853	_	** + **	** + **	** + **	** + **	** + **	+	** + **
*S. typhi* DSM 17058	_	_	+	** + **	** + **	** + **	+	** + **
*S. sonnei* DSM 5570	_	_	+	** + **	** + **	** + **	+	** + **
*S. aureus* ATCC 29737	_	_	+	+	** + **	** + **	+	** + **
Spectrum activity (%)	8/8	3/8	0/8	0/8	0/8	0/8	0/8	0/8
	100	37.5	0	0	0	0	0	0

_ no growth, + positive growth.

### Mode of action for the biosynthesized AgNPs from *A. bisporus*

The biosynthesized AgNPs from *A. bisporus* mode of action against pathogenic bacterial strains is shown in Table 4. Results indicated that the AgNPs have a bactericidal effect with MBC/MIC ≤ 2 toward all the bacterial pathogenic strains of *B. cereus*,* E. faecalis*, and *S. aureus*, *E. coli*, *K. quasipneumoniae*, *P. aeruginosa*, *S. typhi*, and *S. sonnei*. So that the green synthesized AgNPs using *A. bisporus* (Mushroom) inhibit both G^+ve^ and G^−ve^ bacteria in a bactericidal manner.

**Table 4:** MBC and MIC of AgNPs on foodborne pathogenic bacterial strains after incubation at 37 °C for 24 h.

Foodborne pathogen strains	MIC (AgNPs µg/mL)	MBC (AgNPs µg/mL)	MBC/MIC ratio	Mode of action
*B. cereus* ATCC 11,778	500	1000	2	+
*E. faecalis* ATCC 29,212	500	1000	2	+
*S. aureus* ATCC 29,737	250	500	2	+
*E. coli* ATCC 8379	1000	1000	1	+
*K. quasipneumoniae* ATTC 700,603	1000	1000	1	+
*P. aeruginosa* ATCC 27,853	1000	1000	1	+
*S. typhi* DSM 17,058	500	500	1	+
*S. sonnei* DSM 5570	500	500	1	+

Bactericidal ≤ 2, Bacteriostatic ≥ 4.

### Cytotoxicity of biosynthesized AgNPs from *A. bisporus*

**Figure 9:**
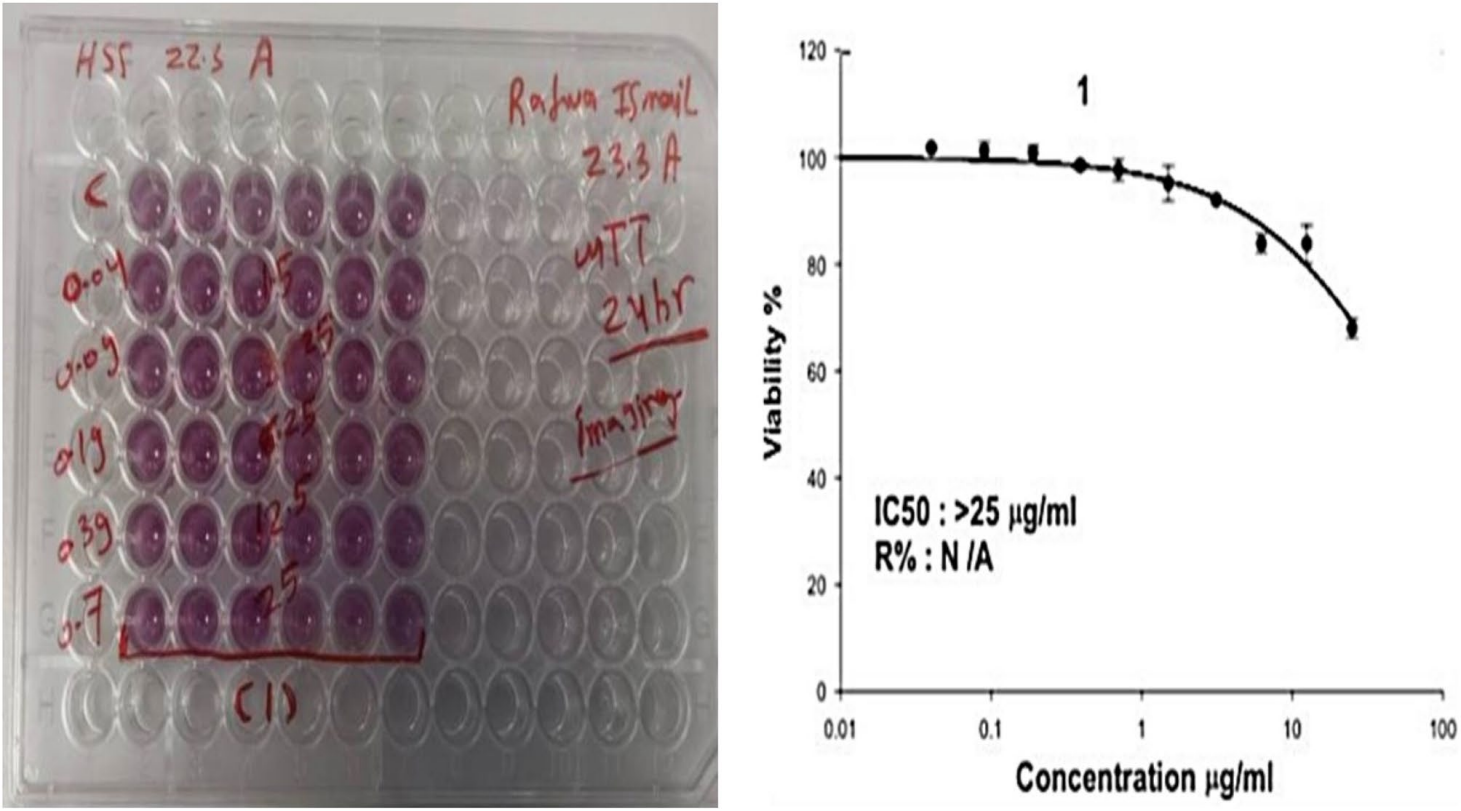
HSF cell viability grown on DMEM medium incubated for 24 h. using different concentrations of AgNPs illustrating that IC_50_ dose is > 25 μg/mL.

Based on the results presented in Fig. 9, the cell viability of HSF cell lines was evaluated at different concentrations of *A. bisporus* AgNPs. The results indicate that the cell viability was relatively high at lower concentrations of AgNPs, with a cell viability of 95.8% observed at a concentration of 0.04 μg/mL. As the concentration of AgNPs increased, the cell viability decreased, with cell viabilities of 91.1%, 89.2%, and 82.3% observed at concentrations of 1.5, 6.25, and 25.0 μg/mL, respectively. The IC_50_ dose of AgNPs was found to be greater than 25 μg/mL, indicating that the AgNPs did not exhibit significant cytotoxicity at the tested concentrations. The log-logistic distributions and functions model curve plotted using the data obtained from the dose–response assay further confirm the low cytotoxicity of *A. bisporus* AgNPs at the tested concentrations. Based on these results, it can be suggested that *A. bisporus* AgNPs have good biocompatibility and could be used at higher concentrations in various applications such as antimicrobial agents, wound healing, anticancer drugs, and food preservation. However, further studies are needed to evaluate the safety and efficacy of *A. bisporus* AgNPs in these applications and investigate their potential toxicity at higher concentrations.

**Figure 10:**
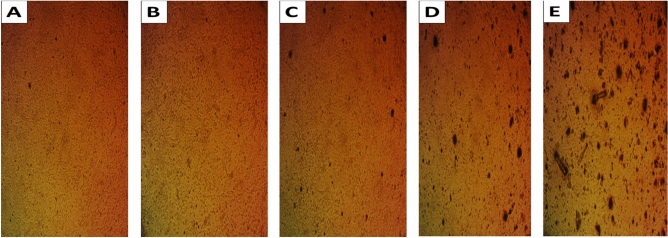
Morphological changes in HSF cell line after treatment with different concentrations of the biosynthesized AgNPs from *A. bisporus*. (**A**) Control sample, (**B**) 0.04 μg/mL, (**C**) 1.5 μg/mL, (**D**) 6.25 μg/mL, (**E**) 25 μg/mL.

The MTT assay is a widely used method to assess the cytotoxicity and cell viability of substances, including AgNPs, against cultured cells. The assay measures the ability of viable cells to reduce MTT to formazan crystals, which can be quantified by measuring the absorbance at 570 nm. The results of the MTT assay can provide valuable information on the potential cytotoxicity of AgNPs towards HSF cells and their potential for use in biomedical applications. The microscopic images presented in Fig. 10a–e illustrate the morphological changes in HSF cells exposed to 0.04 and 25 μg/mL *A. bisporus* AgNPs and negative untreated HSF spherical cell controls. The images show that at concentrations of 0.04 and 25 μg/mL, almost all HSF cells survived relative to the negative control, indicating that *A. bisporus* AgNPs have excellent biocompatibility.

## Discussion

The food sector faces critical problems worldwide due to the development of antibiotic resistance of bacterial pathogens towards chemical control methods. Discovering new alternatives has become a must for facing this important issue. Indeed, the use of fungi in synthesizing NPs leads to producing large amounts of reducing enzymes and proteins rather than using bacteria which catalyze the conversion of metal salts into NPs in short time^1^. Moreover, Fungi have potential cell walls that help in the absorption, reduction of metal ions and production of metal nanoparticles^11^. Many studies exploited nanoparticle (NPs) biosynthesis using edible and therapeutic fungal mushrooms in the last few decades. Also, it was found that mushrooms have the ability to generate both metallic and non-metallic NPs such as gold, silver, ferrous platinum, gold, and selenium^5,8,13,15,37^.

In our study, *A. bisporus* (mushroom) aqueous extract was treated with silver nitrate solution and incubated for 24 h to form AgNPs, which were observed by the color change into brown. Many studies pointed to this color change as well^38^. This color change is due to the both formation of colloidal nanoparticles and excitation of the metal nanoparticles’ SPR through the synthesis process^38^.

To indicate the AgNPs formation, UV–visible spectra was applied and the detected peak’s absorbance value was recorded. As well known, both size and shape of AgNPs reflect the absorption peak apart from the detected peak in the UV spectrum corresponding at wave length of 200-800 nm. In our study, silver surface plasmon band was detected at 456 nm with SPR of 1.5 while the *A. bisporus* extract peak was detected at 265.5 nm with O.D of 1.37. The obtained results showed a shift for absorbance values from the control extract of mushroom compared with the mushroom-Ag preparation, which indicates AgNPs formation. As similar results, the absorption peak of *P. giganteus* extract was located between 400 and 450 nm^24^. Similiarily, the surface plasmon absorption band of SNPs produced by *A. flavus* NJP08 was read at 420 nm using UV–visible and the absorption maxima (λmax) of biosynthesized SNPs was detected in the range of 420 nm in *A. fumigatus*^39^. The main reason for shifting the absorbance values is attributed to the increase or decrease changes in the NP size and SPR peak of metal nanoparticles that is highly dependent on their structure as well as the surrounding chemicals^37,40^.

XRD analysis for AgNPs from *A. bisporus* disclosed the distinctive diffraction peaks of cubic silver’s crystalline planes. Diffraction intense peaks at 2θ values at 38.5°, and 45.5° corresponding to planes (111), and (200), respectively. It belonged to face centered cubic (fcc) crystal type structure and matches with the JCPDS file No. 04-0783. The peak indexed to the (111) plane was more intense which confirms predominant orientation of the AgNPs. This data is further supported by SAED pattern which also confirms that (111) plane is prominent plane as compared to other planes. The broadness of the XRD peaks indicates nano scale of silver particles. The peak spreading of XRD lines at half their maximum intensity is induced by crystallite size, flattening, and micro-strains inside the diffracting domains^24^. Inaddition, the peak intensity along (111) plane is sharper and more prevalent than the other peaks which implies that (111) is the dominant crystallographic plane for AgNPs. It is evident, from the recorded XRD spectrum that no Ag oxide peaks were formed which suggests that biosynthesized AgNPs were well capped by the phytochemicals present in the *A. bisporus* extract. These data are former closely in agreement with the previously reported findings^37^.

DLS is the most used technique for analyzing AgNPs in the colloidal solutions through the reaction of light beams with the biosynthesized NPs^41^.

In our current study, AgNPs mean diameter was 765.7 nm. The metal nanoparticles’ size expresses the interaction of the NPs surface area and the bacterial or fungal species extracts. Usually, the size of AgNPs obtained from DLS investigation was larger than those gained from SEM, TEM and XRD analyses. The obtained results could be attributed to the size assembled from DLS depending on *A. bisporus* extract contains active functional groups which were of the reducing, stabilizing, and capping agents that had accumulated on and sournded the AgNPs surface. Additionally, the AgNPs metallic core and particles polydispersity is related to larger NPs sizes under DLS investigation .By assessing the polydispersity index (PDI) values, DLS analysis provides a detailed characteristic about the homogeneity of the NPs in colloidal solutions. The NPs solution homogeneity is increased or decreased if PDI score is more or less than 0.4, while it considered heterogeneous NPs if the PDI result is equal to or more than 1. The current results indicated that the PDI score of the biosynthesized AgNPs was equal to 0.537^5,8^.

In order to characterize the morphology and size of the synthesized AgNPs, SEM was performed. It shows spherical particles with an average diameter of 67.45–102.7 nm. In line with our current findings^24^, *P. giganteus* mushroom had spherical shaped synthesized AgNPs with diameter of 10 and 85 nm. In addition the shape of the AgNPs was found to be oval and spherical and the size ranges from 500 nm.

FT-IR analysis was used to investigate the presence of active compounds that cap and reduce silver nitrate precursor to form AgNPs. The occurrence of several functional groups in *A. bisporus* extract was indicated the presence of various IR bands. In this study, the IR spectra investigated for the silver nanoparticles revealed the absorption peaks at (a) 2927.76, 3280.58, and 1404.99 cm^−1^ that corresponding to OH group of alcohols and phenols; (b) 1673.52 cm^−1^ and corresponding to C=O stretching of carboxylic acid group; (c) 1032 cm^−1^ which for C–N streching of the (amine) protein. Additionally, Ag was assigned the characteristic bands at 514.79 cm ^−1^.

It was follows that the various fungal strains extracts play a major part in the production and size reduction of Ag into a well-stabilized nano-form^20^.

FTIR analysis found amine bands of the proteins and C–N (amines group), and it is well known that protein–nanoparticle interactions can occur either through free amine groups or cysteine residues in proteins and via the electrostatic attraction of negatively charged carboxylate groups in enzymes^42^. These functional groups are the capping and reducing agents involved in the synthesis of AgNPs, according to the findings of FTIR and HR-SEM–EDX investigation^18^.

The stability of synthesized AgNPs can be estimated using Zeta Potential (ξ) value. It depends on the particles movement under the effect of electric field which confirms the presence and nature of biomolecules adsorbed on the surface of NPs. The magnitude of ξ of NPs gives information about the stability of the bio-synthesized AgNPs. The ξ value recorded for Pt NPs was − 5.7 mV. The negative value of ξ may be due to the capping effect of the NPs by the phytochemicals present in the *A. bisporus* extract and suggests the presence of repulsive forces among the NPs which prevents agglomeration and provides stability to the bio-synthesized NPs. The obtained result was in the line of who stated a negative charge for the nanoparticles formed by *S. aromaticum* extract that due to the capping effect of the NPs by the phytochemicals^43^.

Biosynthesized NPS show high antibacterial activity against different microbial pathogens such as gram-positive and gram-negative bacteria. Our studies illustrated that AgNPs strongly inhibit the growth of *B. cereus*, *E. faecalis,* and *S. aureus*, while it had a medium effect on *P. aeruginosa*,* S. typhi*,* E. coli*,* Sh. sonnei*, and *K. quasipneumoniae*. This activity explained that ions and silver compounds are highly toxic to most bacterial strains. Also, different concentrations of AgNPs of 10, 50, and 100 µl/mL were effective against *E. coli*, *Staphylococcus* sp., *Pseudomonas* sp., and *Bacillus* sp. with IZD of 10.0, 10.0, 8.0 and 14.0 mm with 10.0 µl/mL at 100% AgNPs concentration^44^. In another study of using Gold-Platinum nanoparticles (50 µL) using *Syzygium aromaticum* bud-extract it was found that the IZD was maximum with G-ve bacteria *P. aeruginosa* by 18 mm and G + ve of *S. aureus* with 16 mm.The whole positive net charge of AgNPs has an important role in which Ag^+^ interacts with the negative charge on the bacterial cell wall causing changes in the shape of cell wall morphology and increase permeability of cells that cause liquid leakage of the cell which consequently results in cell death. Moreover, AgNPs binds with the cellular membrane proteins at phosphorous and, sulfur-containing biomolecules as well as interacting with the intracellular components (DNA bases, protein) affecting all metabolic processes (cell division, respiration, survival of the cell). However, Other researches have reported that the Ag^+^, binds with nitrogen and sulfur causing inhibition and disruption for protein structures by binding to thiol and amino groups. It is well known that thiol group is responsible for the induction of reactive oxygen species (ROS), which leads to the inhibition of respiratory enzymes and, consequently, death. In conclusion, Silver ions is a key role as antibacterial agent by interacting with the peptidoglycan in the cell wall and plasma membrane as well as preventing bacterial DNA replication process by the means of interacting with protein sulfhydryl groups^45^.

Additionally, produced AgNPs from used mushroom extract were safe for HSF cells. It records cell viability reached 95.8, 91.1, 89.2, and 82.3% for *A. bisporus* AgNPs concentration of 0.04, 1.5, 6.25, and 25.0 μg/mL, respectively, with IC_50_ doses of > 25 μg/mL AgNPs. In similar studies, biosynthesized AgNPs were safe on HSF cells^9^. So, AgNPS from *A. bisporus* could be used at higher concentrations in many applications such as antimicrobial, wound healing anticancer drugs and food preservation. In similar results, biosynthesized nanoparticles using cell-free extracts mediated, coated, and stabilized by bioactive groups are safer, more biocompatible, and can be applied in biomedical applications^8^. By this study’s end, we recommend further studies on testing AgNPs to biocontrol biofilms in the food industry.

## Conclusion

The use of biological organisms as potential biomechanical tools in the synthesis of AgNPs is a growing area of research in nanobiotechnology. In your study, the AgNPs were synthesized using the *A. bisporus* extract as a reducing and stabilizing agent, which provided a unique and advantageous approach to the synthesis of AgNPs over traditional chemical methods. This approach was found to be easy, non-toxic, and eco-friendly, and the resulting AgNPs exhibited potential antimicrobial activity against all studied bacterial strains. The potential biomedical applications of these AgNPs are significant. They could be used for wound healing, where their antimicrobial properties may help prevent infection and promote healing. They could also be used as antimicrobial agents for different biological purposes and food preservation, where their ability to inhibit bacterial growth could help increase the shelf life of food products. The emplying of biosynthesized nanoparticles with functional properties, such as the AgNPs synthesized in your study, holds great promise for large-scale production and can shape the future of nanobiotechnology research. The data from your study suggest that the AgNPs synthesized using the *A. bisporus* extract have potential for use in medicine and other applications, and further research in this field may lead to the development of new and innovative nanomaterials with a wide range of potential applications.

## Data Availability

The raw data and analyzed data used during the current study available from the corresponding author on reasonable request. All microbial pathogens were provided by Agricultural Microbiology Department, Faculty of Agriculture, Ain Shams University, Cairo, Egypt and was deposited in the following strain providers (1) *B. cereus* ATCC 11,778 was from the ATCC collection https://www.atcc.org/products/11778. (2) *E. fecalis* ATCC 29,212 was from the ATCC collection https://www.atcc.org/products/29212. (3) *S. aureus* ATCC 29,737 was from the ATCC collection https://www.atcc.org/products/29737. (4) *E. coli* ATCC 8379 was from ATCC collection https://www.atcc.org/products/8379 and was deposited in GenBank with taxonomy ID: NCBI:txid 481,805 https://www.ncbi.nlm.nih.gov/Taxonomy/Browser/wwwtax.cgiAgNPs id = 481,805. (5) *K. pneumonia* ATCC 700,603 was from ATCC collection https://www.atcc.org/products/700603. (6) *P. aeruginosa* 27,853 was from ATTC collection https://www.atcc.org/products/27853. (7) *S. typhi* DSM 17,058 was from DSM collection https://www.dsmz.de/collection/catalogue/details/culture/DSM-17058. (8) *S. sonii* DSM 5570 was from DSM collection https://www.dsmz.de/collection/catalogue/details/culture/DSM-5570.
